# Hydrogenotrophic methanogenesis is the key process in the obligately syntrophic consortium of the anaerobic ameba *Pelomyxa schiedti*

**DOI:** 10.1038/s41396-023-01499-6

**Published:** 2023-08-26

**Authors:** Sebastian C. Treitli, Pavla Hanousková, Vladimír Beneš, Andreas Brune, Ivan Čepička, Vladimír Hampl

**Affiliations:** 1 Department of Parasitology, Faculty of Science, Charles University, BIOCEV, Průmyslová 595, 252 42 Vestec, Czech Republic; 2 Department of Zoology, Faculty of Science, Charles University, Viničná 7, 128 00 Prague 2, Czech Republic; 3 Genome Biology Unit, European Molecular Biology Laboratory (EMBL), Heidelberg, Germany; 4 RG Insect Gut Microbiology and Symbiosis, Max Planck Institute for Terrestrial Microbiology, Marburg, Germany

**Keywords:** Symbiosis, Microbial ecology, Bacterial genetics

## Abstract

*Pelomyxa* is a genus of anaerobic amoebae that live in consortia with multiple prokaryotic endosymbionts. Although the symbionts represent a large fraction of the cellular biomass, their metabolic roles have not been investigated. Using single-cell genomics and transcriptomics, we have characterized the prokaryotic community associated with *P. schiedti*, which is composed of two bacteria, *Candidatus* Syntrophus pelomyxae (class *Deltaproteobacteria*) and *Candidatus* Vesiculincola pelomyxae (class *Clostridia*), and a methanogen, *Candidatus* Methanoregula pelomyxae. Fluorescence in situ hybridization and electron microscopy showed that *Ca*. Vesiculincola pelomyxae is localized inside vesicles, whereas the other endosymbionts occur freely in the cytosol, with *Ca*. Methanoregula pelomyxae enriched around the nucleus. Genome and transcriptome-based reconstructions of the metabolism suggests that the cellulolytic activity of *P. schiedti* produces simple sugars that fuel its own metabolism and the metabolism of a *Ca*. Vesiculincola pelomyxae, while *Ca*. Syntrophus pelomyxae energy metabolism relies on degradation of butyrate and isovalerate from the environment. Both species of bacteria and the ameba use hydrogenases to transfer the electrons from reduced equivalents to hydrogen, a process that requires a low hydrogen partial pressure. This is achieved by the third endosymbiont, *Ca*. Methanoregula pelomyxae, which consumes H_2_ and formate for methanogenesis. While the bacterial symbionts can be successfully eliminated by vancomycin treatment without affecting the viability of the amoebae, treatment with 2-bromoethanesulfonate, a specific inhibitor of methanogenesis, killed the amoebae, indicating the essentiality of the methanogenesis for this consortium.

## Introduction

The genus *Pelomyxa* belongs to Archamoebae and its representatives inhabit hypoxic freshwater environments [1–3]. Species of *Pelomyxa*, which may reach several millimeters in size, possess numerous non-motile flagella with an aberrant axonemal structure [4, 5] and were originally considered to lack elementary cell organelles such as Golgi bodies and mitochondria. Early morphological studies of *Pelomyxa palustris* have shown the presence of microbody-like granules that might represent a mitochondria-related organelle (MRO) [6], and in *Pelomyxa schiedti* such MRO was recently characterized using single-cell genomic and transcriptomic approaches [7]. Identification of the MROs in representatives of *Pelomyxa* by transmission electron microscopy is difficult because these amoebae harbor a variety of bacterial endosymbionts [2, 8–11]. These endosymbionts differ in size and localization, are positioned either inside vacuoles or freely in the cytoplasm, some tending to localize around the nuclei [2, 10]. Already the original description of *P. palustris* mentions small “dancing rods” that were observed when the cells were broken [12]. Later studies suggested that *P. palustris* harbored three types of endosymbionts; Gram positive and Gram negative slender rods and a larger Gram positive bacterium with a rectangular shape [13, 14]. Based on the production of methane by cultures of *P. palustris* and the characteristic autofluorescence of F_420_ coenzyme observed in the two slender morphotypes, it was suggested that these might be methanogens [15]. This was further supported by the isolation of a slender Gram positive methanogenic archaeon from *P. palustris* into a pure culture where it grew on H_2_/CO_2_ and formate and it was identified as *Methanobacterium formicicum* [16]. However, the reliance on the F_420_ fluorescence is not fully conclusive, as this cofactor is also produced by certain actinobacteria [17]. Moreover, these observations were in conflict with other studies on *P. palustris*, which claimed that the third morphotype, represents the methanogenic symbionts [18]. The situation was finally clarified using 16 S rRNA gene sequencing and fluorescence in situ hybridization (FISH), which identified the large rectangular-shaped cells located around the nucleus as a methanogenic archaeon from the genus *Methanosaeta*, the Gram negative rods as deltaproteobacteria from genus *Syntrophorhabdus*, and the Gram positive rods as actinobacteria from the genus *Rhodococcus* [19]. Although the metabolism of the endosymbionts remained unknown, it was suggested that the microbial community inside *P. palustris* resembled communities found in activated sludge [19], where members of the genus *Rhodococcus* and *Syntrophorhabdus* occur in syntrophic associations with methanogens [20, 21].

To characterize the prokaryotic symbionts and disentangle the metabolic bonds between the community members, we used the available single-cell genomic data for *P. schiedti* [7] to identify its endosymbionts and to assemble their genomes. By generating custom single-cell transcriptomes aimed to also capture the prokaryotic mRNA, we characterized the major biochemical processes contributed by each partner and provide evidence that the methanogen plays a key role in this complex quadripartite symbiosis.

## Materials and methods

### Cell culture and manipulation

Polyxenic cultures of *Pelomyxa schiedti* strain SKADARSKE were maintained in Sonneborn’s *Paramecium* medium [22] and transferred every two weeks as described previously [2]. For removal of the symbionts, the cultures were treated with vancomycin (Sigma-Aldrich) at a final concentration of 40 µg/ml or with 2-bromoethanesulfonate (BES) (Sigma-Aldrich) a final concentration of 5 mM. In the case of the treated cultures, the cells were transferred weekly.

### Sample preparation and FISH

Several tubes of *Pelomyxa schiedti* were merged and pelleted by centrifugation at 600 × *g* for 10 min. The pelleted cells were resuspended in Trager’s Solution U [23], and samples were fixed on ice for six hours in 4% formaldehyde. Probes for FISH were designed using ARB 6.0.4 [24] and the Silva REF NR 99 SSU database release 126 [25]. FISH preparations were performed according to the protocol described elsewhere [26]. Detailed procedures for cell fixation and FISH are provided in Supplemental materials and methods.

### Transcriptome amplification

Transcriptome sequencing was performed based on a modified SmartSeq2 protocol [27] designed to capture also the bacterial transcripts. The modifications include bacterial cell lysis either using rLysozyme or Mutanolysin, polyadenylation of the transcripts using PolyA polymerase, and blocking of the adenylation of rRNA using the EMBR-seq strategy [28]. Detailed procedures for single-cell transcriptome amplification, library preparation, and analysis are provided in Supplemental materials and methods.

### Genome assembly, binning, and annotation

The single-cell genome assemblies were generated from seven single-cell genomic libraries of *P. schiedti* (NCBI BioProject PRJNA672820) [7]. Each single-cell genome was assembled using SPAdes 3.13.0 [29]. Binning of contigs was performed using tetraESOM [30] and their completeness was estimated using CheckM [31]. Prokaryotic genomes were annotated using Prokka 1.14.6 [32] and EggNOG-mapper [33, 34]. All annotations were merged in a single GenBank file using emapper2gbk [35] and imported into Pathway Tools v 23.0 [36] for further curation and analysis. For all pathways of interest, the annotations were manually curated. Detailed procedures for binning and annotation are provided in Supplemental materials and methods.

### Phylogenetic analysis

Several 16 S rRNA gene datasets were composed including the prokaryotic symbionts of *Pelomyxa palustris* [19] where possible. The sequences were aligned using MAFFT 7 [37] with the G-INS-i algorithm followed by manual inspection and trimmed using BMGE [38] with default parameters. Maximum Likelihood (ML) phylogenetic trees were constructed using IQ-TREE 1.6.12 [39] with the Model Finder Plus setting and with 1000 non-parametric bootstrap replicates. The dataset for phylogenomic analyses was created with GTDB-Tk [40] and the GTDB database [41]. A ML phylogenetic tree was inferred by IQ-TREE 1.6.12 using the Posterior Mean Site Frequency (PMSF) empirical model with a LG + F + G guide tree. The branch supports were estimated using the ultrafast bootstrapping with 10000 replicates. Detailed procedures about the phylogenetic analyses are provided in the Supplemental materials and methods.

## Results

### Species composition of the *Pelomyxa schiedti* endosymbiont community

**Fig. 1:**
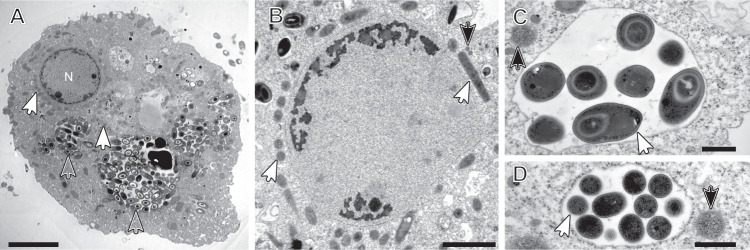
Transmission electron micrographs of *Pelomyxa schiedti* cells. **A** Overview of *P. schiedti* cell with the nucleus (N); the different arrowheads indicate different prokaryotic morphotypes; Open arrowheads point to putative *Ca*. Vesiculincola pelomyxae cells, while white filled arrowheads point to putative *Ca*. Methanoregula pelomyxae cells. **B** Detail of the nucleus and the bacterial symbionts located around it; white arrowheads point to putative *Ca*. Methanoregula pelomyxae cells while black filled arrowheads point to putative MROs. **C**, **D** Detail of the bacterial symbionts located in the cytosol (black filled arrowheads) and inside the vacuole (white arrowheads). The bacterial cells in the vacuole (**C**) are in the process of endospore formation; the diameter of the sporangia is considerable larger than that of the vegetative cells (**D**) The black filled arrowheads in C and D point to putative *Ca*. Syntrophus pelomyxae cells. Scale bar: A – 5 µm; B – 2 µm; C, D – 500 nm.

Electron microscopy (EM) observations of *Pelomyxa schiedti* confirmed that the cell harbors numerous prokaryotic symbionts with different morphotypes [2] (Fig. 1). One of the morphotypes is enclosed in vesicles (Fig. 1A, C, D), the others occur freely in the cytosol. (Fig. 1A, B, D). We re-assembled the published single-cell metagenomes of *P. schiedti* [7] and after the removal of eukaryotic contigs, binned the data using tetraESOM [30]. Each assembly contained multiple bins of prokaryotes, but for further analyses we focused on the five bins present in all samples, which were classified as *Ca*. Methanoregula pelomyxae, *Ca*. Syntrophus pelomyxae, *Ca*. Vesiculincola pelomyxae, an *Acetomicrobium* sp., and a member of *Victivalles*.

**Fig. 2:**
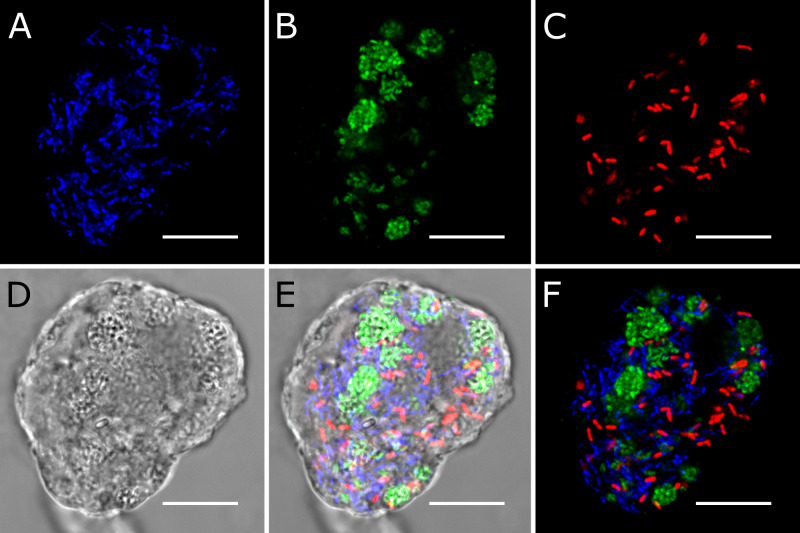
FISH of *Pelomyxa schiedti* cells. **A** Probe Meth-P-972 specific for *Ca*. Methanoregula pelomyxae; **B** Probe Rum-P-276 specific for *Ca*. Vesiculincola pelomyxae; **C** Probe SYN-P-182 specific for *Ca*. Syntrophus pelomyxae; **D** DIC view of the cell; **E** Merged picture of **A**, **B**, **C** and **D**; **F** Merged picture of **A**, **B** and **C**; All scale bars: 10 µm.

We designed specific oligonucleotide probes against the 16 S rRNA gene of each metagenomic bin (Table S1). FISH with probes against *Ca*. Methanoregula pelomyxae (Meth-P-972), *Ca*. Syntrophus pelomyxae (Syn-P-182), and *Ca*. Vesiculincola pelomyxae (Rum-P-276) showed clear signals in all *P. schiedti* cells (Fig. 2). *Ca*. Methanoregula pelomyxae are slender rods and quite numerous (Fig. 2A), particularly around the nuclei (Supplementary video S1), as evidenced also by EM (Fig. 1B). Cells of *Ca*. Vesiculincola pelomyxae are the most abundant and aggregate in clumps (Fig. 2B); their coccoid shape, the formation of terminal endospores, and their enclosure in membrane vesicles is evidenced by EM (Fig. 1C, D). Cells of *Ca*. Syntrophus pelomyxae, the least abundant of the three endosymbionts, are rod shaped (Fig. 2C) and evenly distributed throughout the cytosol. Probes targeting the *Victivalles* and *Acetomicrobium* sp. did not yield any signal; they were considered contaminants and not analyzed further.

We evaluated the essentiality of each endosymbiont by treating the cultures of *P. schiedti* with vancomycin, an antibiotic that inhibits bacterial cell wall biosynthesis, or 2-bromoethanesulfonate (BES), a specific inhibitor of methanogenesis. Vancomycin treatment (Fig. S1) removed *Ca*. Vesiculincola pelomyxae from most host cells within six weeks as demonstrated using FISH (Fig. S1B). After 16 weeks, both *Ca*. Vesiculincola pelomyxae and *Ca*. Syntrophus pelomyxae had disappeared entirely (Fig. S1G, H), but the cells, now containing only *Ca*. Methanoregula pelomyxae, remained viable. By contrast, cultures treated with BES died within seven days, suggesting that the methanogen is an essential endosymbiont.

### Metabolism of *Pelomyxaschiedti*

**Fig. 3:**
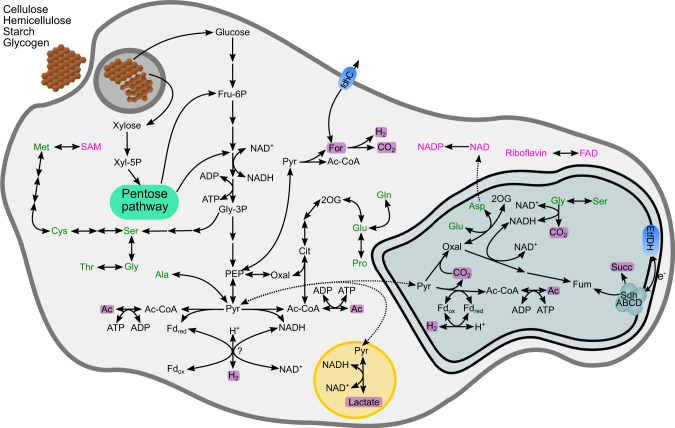
Metabolic map of *Pelomyxa schiedti* reconstructed from the draft genome. Amino acids are shown in green; vitamins and cofactors are shown in pink. Non-standard abbreviations: 2OG 2-oxoglutarate, Ac Acetate, Ac-CoA acetyl-coenzyme A, Cit citrate, EtfDH electron-transferring flavoprotein dehydrogenase, For formate, Fru-6P fructose-6-phosphate, Fum fumarate, Gly-3P glycerate 3-phosphate, Oxal oxaloacetate, PEP phosphoenolpyruvate, Pyr pyruvate, SAM S-adenosylmethionine, SdhABCD Succinate dehydrogenase complex, Succ succinate, Xyl-5P xylose 5-phosphate. Putative metabolic end products are highlighted with a purple background. The double membrane compartment represents the MRO while the yellow one represents the peroxisome.

A previous study on the *P. schiedti* genome had reconstructed the metabolic pathways in its anaerobic peroxisomes and MROs resembling hydrogenosomes [7]. Here, we used the same dataset to reconstruct the energy metabolism of *P. schiedti*. The genome encodes 182 glycosyl hydrolases (GH) classified into 44 families that are putatively involved in the digestion of polysaccharides (Dataset 1). The most abundant families GH13 (19 genes), GH3 (15 genes), and GH5 (9 genes) encode α-amylases, β-glucosidases, and endoglucanases that are involved in degradation of cellulose, hemicelluloses, starch, and glycogen, generating simple sugars that are funnelled into glycolysis (Fig. 3). From the glycolytic intermediates, *P. schiedti* can synthesize de novo 10 out of the 20 proteinogenic amino acids and various cofactors (Fig. 3). Since no pathway for de novo synthesis of nucleotides is present, *P. schiedti* most likely scavenges and recycles nucleotides either from food or environment. We also identified all enzymes required for the biosynthesis and β-oxidation of fatty acids (Dataset 2).

The NADH formed during glycolysis and β-oxidation can be reoxidized by lactate dehydrogenase (LDH), which reduces pyruvate to lactate without ATP production. This process putatively takes place in the anaerobic peroxisomes, as only one copy of LDH was identified in the genome, and the enzyme has been shown to be localized in the peroxisomes [7]. In the cytosol, pyruvate can be oxidized to acetyl-CoA either by pyruvate:ferredoxin oxidoreductase (PFOR) or pyruvate:NADP+ oxidoreductase (PNO). This “extended glycolysis” allows subsequent production of an additional ATP by acetate synthase, but at the same time produces additional reduced cofactors (ferredoxin or NADH). Finally, pyruvate-formate lyase (PFL) can convert pyruvate to acetyl-CoA and formate. Since the latter can be cleaved to CO_2_ and H_2_ by formate hydrogenlyase (Pelo_6878), it would not generate any additional reducing equivalents but also not contribute to the reoxidation of NADH from glycolysis.

The genome of *P. schiedti* encodes eight hydrogenases, which were classified by their domain structure in two groups: “short” hydrogenases containing only an [FeFe]-hydrogenase domain, and “long” hydrogenases containing an N-terminal NuoG domain followed by an [FeFe]-hydrogenase, and a C-terminal CysJ domains. Two short and one long hydrogenase were predicted to be localized in the MRO [7], while the remaining five long hydrogenases are putatively localized in the cytosol. Their CysJ domain contains an NADH binding pocket, and phylogenetic analysis places them in a well-supported clade of other eukaryotic and NADH-dependent bacterial hydrogenases (Fig. S2). The cytosolic hydrogenases may be involved in reoxidation of NADH and ferredoxin, which would allow an extended glycolysis yielding additional ATP. At the same time, a fraction of the extended glycolysis likely takes place in the MRO, where the redox balance is maintained by short hydrogenases and by pyruvate carboxylation and reduction to succinate [7] (Fig. 3).

### Genome and metabolism of *Ca*. Vesiculincola pelomyxae

The genome assembly of *Ca*. Vesiculincola pelomyxae consists of 17 scaffolds with a total length of ~1.4 Mbp, a GC content of 33.1%, and a genome completeness of 85.2% (Table 1). We identified 47 tRNA genes, two ribosomal RNA operons and predicted 1301 partial or complete putative coding sequences. Phylogenetic analysis of the 16 S rRNA gene failed to position the bacterium in any known genera with strong support (Fig. S3). Phylogenomic analysis with GTDB-Tk, using an alignment of 120 protein-coding genes, showed that *Ca*. Vesiculincola pelomyxae is most closely related to a clade of uncultured bacteria in the “Acutalibacteriaceae” family (Fig. S4), whose members are mostly associated with vertebrate feces [42]. The genome sizes of *Ruminococcus bromii* (still classified in the family *Oscillospiraceae* under the ICNP), the most closely related described species, is considerably larger (2.2 Mbp). Based on average relative evolutionary distance to *R. bromii* and other taxa (0.89), *Ca*. Vesiculincola pelomyxae represents a new genus-level lineage.

**Table 1:** General features of the genomes obtained in this study.

Sample	Scaffolds	Total length (bp)	N50 (kbp)	G + C content (%)	Putative CDS	Completeness (%)	Contamination (%)
*Ca*. Vesiculincola pelomyxae	17	1,409,312	342	33.1	1301	85.2	0
*Ca*. Syntrophus pelomyxae	84	4,973,966	256	49.9	4484	97.4	7.4
*Ca*. Methanoregula pelomyxae	1	1,949,073	1949	54.8	1991	99.7	0

**Fig. 4:**
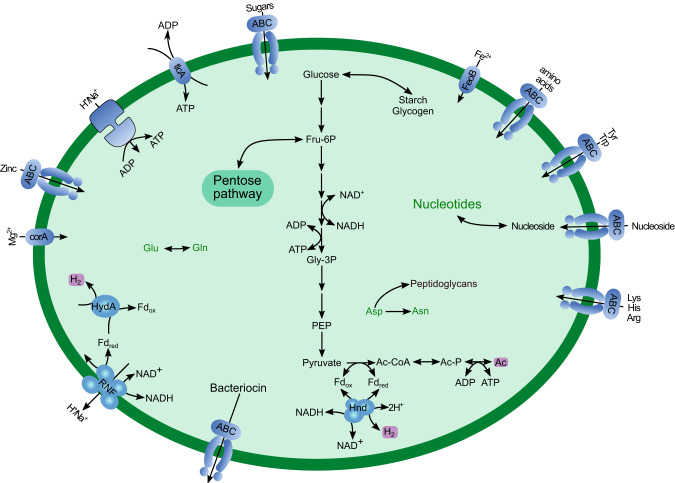
Metabolic map of *Ca*. Vesiculincola pelomyxae reconstructed from the draft genome. Non-standard abbreviations: Ac Acetate, Ac-CoA acetyl-coenzyme A, Ac-P acetyl-phosphate, Fd ferredoxin, Fru-6P fructose-6-phosphate, Gly-3P glycerate 3-phosphate, PEP phosphoenolpyruvate. Putative metabolic end products are highlighted with a purple background.

The genome encodes enzymes involved in starch degradation and an ABC transporter of unknown specificity involved in carbohydrate translocation. The strain possesses a full glycolytic pathway and extended glycolysis. A canonical phosphate acetyltransferase (pta) was absent, but we identified a phosphotransacylase PduL (Dataset 3), which has been shown to substitute for pta in the conversion of acetyl-CoA to acetyl-phosphate required for the subsequent substrate-level phosphorylation by acetate kinase [43, 44]. A nucleotide triphosphate transport protein (NTT) is encoded in the genome (Dataset 3), suggesting that ATP could be also obtained from the host. Enzymes producing reduced fermentation products were not detected, including the LDH present in other representatives of the family “Acutalibacteraceae”, such as *Ruminococcus bromii* and *Clostridium leptum*. However, the genome encodes an electron-confurcating hydrogenase (HndABC) of group A1, which would allow the concomitant reoxidation of NADH and reduced Fd, provided that the H_2_ is maintained at low partial pressure. An Rnf complex and a V-type ATPase probably serve to adjust redox balance and generate a membrane potential. The absence of pathways for the de novo biosynthesis of amino acids and nucleotides is compensated by a complex array of ABC transporters for the uptake of amino acids and nucleosides (Fig. 4).

Genes involved in spore formation and germination were highly expressed (Dataset 3). This is in agreement with the abundance of sporulating cells observed by EM (Fig. 1A, C) and the observation that *R. bromii* and *C. leptum* are important endospore formers among the human gut microbiota [45]. The transcriptome contained several transcripts of small proteins that were identified as bacteriocins by manual annotation. Subsequent searches using BAGEL 4 [46] identified two operons for the synthesis of bacteriocins, which were both highly expressed (Dataset 3).

### Genome and metabolism of *Ca*. Syntrophus pelomyxae

**Fig. 5:**
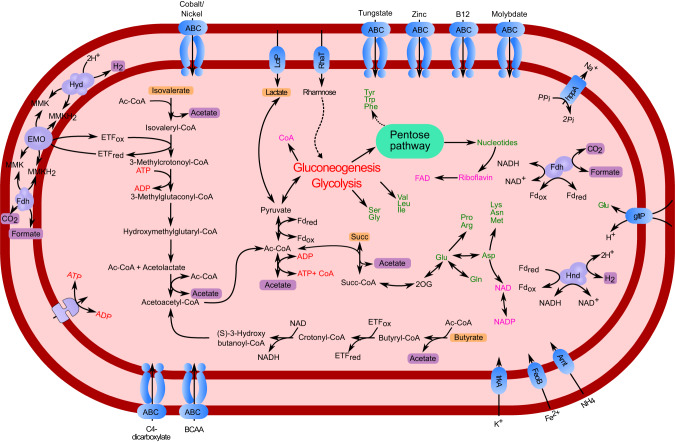
Metabolic map of *Ca*. Syntrophus pelomyxae reconstructed from the draft genome. Amino acids are shown in green; vitamins and cofactors are shown in pink. Non-standard abbreviations: 2OG 2-oxoglutarate, Ac-CoA acetyl-coenzyme A, CoA coenzyme A, BCAA Branched chain amino acids, ETF Electron transfer flavoprotein, MMK Methylmenaquinone, Succ succinate, Succ-CoA succinate coenzyme A. Putative metabolic end products are highlighted with a purple background. Metabolites that are putatively used from the host and/or environment are highlighted with an orange background.

The genome assembly of *Ca*. Syntrophus pelomyxae consists of 84 scaffolds with a total length of ~4.97 Mbp, a GC content of 49.9% and a genome completeness of 97.4% (Table 1). A total of 4484 complete and partial coding sequences were predicted in the genome (Table 1). Phylogenetic analysis of the 16 S rRNA gene identified the bacterium as member of the genus *Syntrophus* (*Deltaproteobacteria*) (Fig. S5). A reconstruction of its metabolism based on the annotation of coding sequences and their expression levels in transcriptomic analyses suggests that the symbiont derives its ATP mainly from the fermentation of isovalerate and butyrate to acetate (Fig. 5). In both pathways, the substrates are converted to acetyl-CoA by β-oxidation, followed by the subsequent production of ATP. The oxidation steps involve an electron-transferring flavoprotein (ETF), which transfers its reducing equivalents to methyl-menaquinone (MMK) via an membrane-bound ETF:MMK oxidoreductase complex [47, 48]. Subsequently, MMK is oxidized either by membrane-bound formate dehydrogenase (Fdh) or hydrogenase complexes (Fig. 5). Under standard conditions, the fermentation of butyrate and isovalerate to acetate and H_2_ is thermodynamically unfavorable, but it becomes exergonic at low hydrogen partial pressure [49, 50]. Enzymes that would allow *Ca*. Syntrophus pelomyxae to use other electron acceptors like fumarate, oxygen, or sulfate, as found in other representatives of *Syntrophobacterales* [51], were not present.

We also identified in the genome an L-rhamnose:proton symporter (CSYNP_04080) and a lactate permease (CSYNP_01065), both of which are expressed (Dataset 4). It is unclear how the rhamnose is funnelled into the metabolism, as we did not identify any enzymes involved in rhamnose degradation. However, the presence of a lactate racemase and several lactate dehydrogenases, which are both expressed at low levels, suggests that lactate might be a substrate for syntrophic oxidation (Fig. 5).

The genome of *Ca*. Syntrophus pelomyxae encodes most of the enzymes required to synthesize 16 out of the 20 amino acids, all nucleotides as well as several cofactors (Fig. 5). Based on the transcriptome read abundances, we hypothesize that the entry into the biosynthetic pathways is either via acetyl-CoA, which is shuttled into gluconeogenesis via PFOR, or via succinate, which is converted into 2-oxoglutarate and further to glutamate (Fig. 5) that can serve as substrate for synthesis of various other amino acids or cofactors.

### Genome and metabolism of *Ca*. Methanoregula pelomyxae

The genome assemblies of *Ca*. Methanoregula pelomyxae from individual *P. schiedti* cells were composed of 2–14 scaffolds. We aligned these assemblies using MAUVE [52] and designed specific primers to amplify the missing ~8 kbp fragment to circularize the chromosome. The final assembly size was ~1.95 Mbp, a GC content of 54.8% with 1991 predicted coding sequences and a genome completeness of 99.7% (Table 1). Phylogenetic analysis of the 16 S rRNA gene placed the endosymbiont in the genus *Methanoregula* (*Methanomicrobiales*) (Fig. S6).

**Fig. 6:**
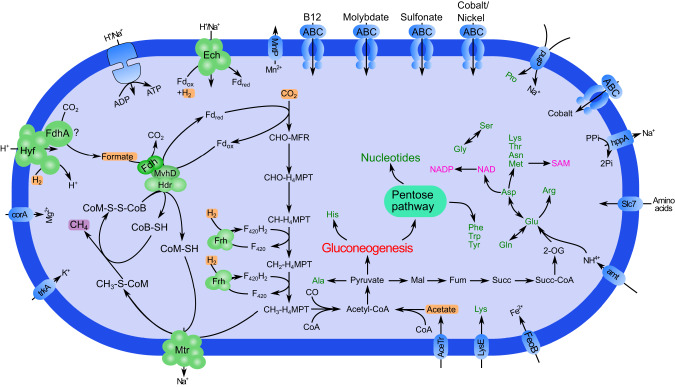
Metabolic map of *Ca*. Methanoregula pelomyxae reconstructed from the draft genome. Amino acids are shown in green; vitamins and cofactors are shown in pink. Non-standard abbreviations: 2OG 2-oxoglutarate, CH_3_-S-CoM methyl coenzyme M, CHO-MFR formylmethanofuran, CO carbon monoxide, H_4_-MPT Tetrahydromethanopterin, CH-H_4_MPT 5,10-Methenyltetrahydromethanopterin, CH_2_-H_4_MPT 5,10-Methylenetetrahydromethanopterin, CH_3_-H_4_MPT 5-Methyltetrahydromethanopterin, CoA coenzyme A, CoB-SH Coenzyme B, CoM-SH Coenzyme M, CoM-S-S-CoB Coenzyme M 7-mercaptoheptanoylthreonine-phosphate heterodisulfide, Fum fumarate, Mal malate, MFR methanofuran, SAM S-adenosylmethionine, Succ succinate, Succ-CoA succinate coenzyme A. Putative metabolic end products are highlighted with a purple background. Metabolites that are putatively used from the host and/or environment are highlighted with an orange background.

The predicted metabolism of *Ca*. Methanoregula pelomyxae resembles a typical hydrogenotrophic methanogen that uses H_2_ and formate to reduce CO_2_ to methane (Fig. 6). The pathway of methanogenesis is essentially the same as in other hydrogenotrophic methanogens. The first step is the reduction of CO_2_ to formylmethanofuran, which is driven by the reduced ferredoxin produced by a soluble heterodisulfide reductase (HdrABC), and the reduced cofactor F_420_ required for the reduction of methenyltetrahydromethanopterin (CH-H_4_MPT) and methylene-tetrahydromethanopterin (CH_2_-H_4_MPT) is provided by a F_420_-reducing hydrogenase (Frh) [53]. The most abundant reads in the transcriptome are methylene-tetrahydromethanopterin dehydrogenase (Mtd) and F_420_-reducing hydrogenase (Frh) (Dataset 5).

The formation of reduced ferredoxin during the reduction of the heterodisulfide of coenzyme M (CoM) and coenzyme B (CoB) that is formed in the last step of methanogenesis, however, is unusual. Like other members of *Methanomicrobiales*, *Ca*. Methanoregula pelomyxae lacks a canonical MvhABGD hydrogenase [54]. Instead, the electron-bifurcating complex most likely gets its electrons from FdhAB, which is linked to HdrABC by an MvhD subunit (Fig. 6). It has been shown that VhuD (a homolog of MvhD) can interact with Fdh in *Methanococcus maripaludis* [55]. However, the absence of a formate transporter remains puzzling, because the closely related *Methanoregula boonei*, which lacks this transporter as well, is unable to use formate as a substrate for methanogenesis [56, 57]. As in all hydrogenotrophic methanogens, ATP is generated via a V-Type ATP synthase, using the electrochemical Na^+^ gradient formed by the CH_3_-H_4_MPT: CoM methyltransferase (Mtr) complex [58].

Like other Methanomicrobiales, *Ca*. Methanoregula pelomyxae has a CO dehydrogenase/acetyl-CoA synthase complex that allows the production of acetyl-CoA. At the same time an acetate transporter and an acetyl-CoA synthetase can be found in the genome. This indicates that acetyl-CoA is the main entry point into the assimilatory metabolism. The genome encodes the biosynthetic pathways for 13 amino acids, transporters for the uptake of lysine, proline, and other amino acids (Fig. 6). We also identified pathways for de novo biosynthesis of nucleotides via gluconeogenesis and pathways for biosynthesis of various cofactors (Fig. 6).

## Discussion

Combining the single-cell genomic data of *P. schiedti* [7] with the newly generated single-cell transcriptomes and 16 S rRNA FISH allowed us to characterize the quadripartite endosymbiotic consortium of *P. schiedti*, reconstruct the metabolism of each member, and deduce the roles of the ameba and its symbionts. The consortium is composed of a methanogen, *Ca*. Methanoregula pelomyxae, a deltaproteobacterium, *Ca*. Syntrophus pelomyxae, and a firmicute, *Ca*. Vesiculincola pelomyxae.

**Fig. 7:**
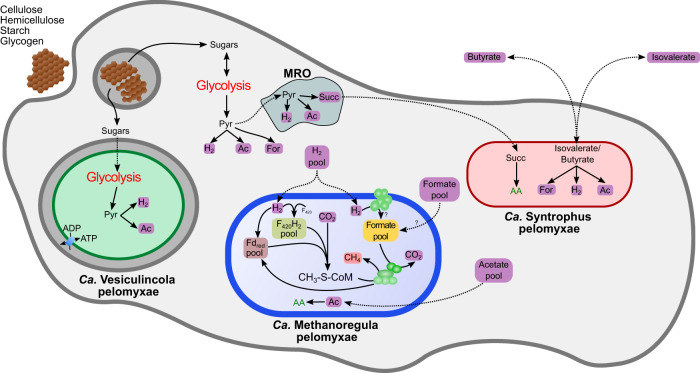
Schematic view of the proposed interaction between the bacterial endosymbionts and *P. schiedti*. The eukaryote uses a complex array of GHs to digest starch, cellulose and glycogen generating monosaccharides. The eukaryote ferments these sugars to acetate (Ac) H_2_ and formate (For). Sugars are also used by *Ca*. Vesiculincola pelomyxae that ferments them to acetate (Ac) and H_2_. *Ca*. Syntrophus pelomyxae uses isovalerate, butyrate and succinate (Succ) to fuel its metabolism. Isovalerate and butyrate is most probably taken from the environment, while succinate is obtained from the host. The host with the two bacteria generates a H_2_, acetate and formate pool that is used by the methanogen for methanogenesis and biosynthesis, keeping the overall concentration of the fermentation products low to facilitate the metabolic flux in the other community members. The question marks indicate the uncertainty of how the formate gets imported by the methanogen, or if is produced using the formate hydrogenlyase and FdhA. AA amino acids, CH_3_-S-CoM methyl coenzyme M, F_420_H_2_ reduced coenzyme F_420,_ Fd_red_ reduced ferredoxin.

The results of genome annotations and transcriptomic analysis allowed us to present the first hypothesis on the metabolic interactions in the consortium (Fig. 7). The external carbon sources are polysaccharides (cellulose, hemicellulose, starch, and glycogen), which are phagocytized and depolymerized by *P. schiedti*. This is not an unusual feature for Archamoebae, as the *Mastigamoeba balamuthi* genome also encodes enzymes to degrade celluloses and hemicelluloses [59]. The monomeric sugars fuel the fermentative metabolism of the ameba, which consists of an extended glycolysis that occurs in the cytosol and partially in the MRO [7]. In the cytosol, the pyruvate is converted to acetate, CO_2_, H_2_, and possibly formate (Fig. 3).

To maintain the redox balance and to maximize ATP production, the ameba reoxidizes NADH and reduced ferredoxin using long hydrogenases, which are characterized by an N-terminal NuoG domain, a [FeFe] hydrogenase domain, and a C-terminal CysJ domain, the latter containing an NADH binding pocket. Similar hydrogenases have been identified in *Trichomonas vaginalis* (Parabasalia) [60], *Stygiella incarcerata* [61], and *Pygsuia biforma* [62]. Phylogenetic analysis places them in a clade of bacterial NADH-dependent [FeFe] hydrogenases of group A6 (Fig. S2) It is possible that the long hydrogenases are electron-confurcating hydrogenases involved in the simultaneous reoxidation of reduced ferredoxin and NADH. However, the additional 4Fe-4S iron sulfur cluster near the C terminus (or in the beta subunit) characteristic for such enzymes [63] was not found in any hydrogenase of *P. schiedti*. Therefore, it is more likely that they use only NADH as electron donor, which would make the H_2_ formation extremely sensitive to hydrogen partial pressure. This would require a second ferredoxin-dependent hydrogenase to reoxidize the cytosolic ferredoxin. A potential candidate is one of the short hydrogenases (Pelo_12595), whose location in the MRO remains to be proven experimentally [7]. It is also possible that most of the pyruvate in the cytosol is converted to acetyl-CoA by PNO without production of reduced ferredoxin. In either case, experimental evidence is required to establish the substrates for these hydrogenases and elucidate the redox balance, which may also involve the anaerobic peroxisomes of the ameba [7].

A reoxidation of NADH by LDH would decrease the proportion of pyruvate available for ATP production in the cytosol, and potentially starving the MRO. On the other hand, the reoxidation of NADH by H_2_ formation requires low hydrogen partial pressure [63, 64]. This may explain why *Ca*. Methanoregula pelomyxae is indispensable for the ameba due to its crucial role as a hydrogen scavenger. The potential dependence of *Ca*. Methanoregula pelomyxae on formate for heterodisulfide reduction (Fig. 6) is enigmatic, especially in the absence of a formate transporter. A possible solution would be the internal production of formate by a second, F_420_-dependent, formate dehydrogenase as it has been shown for *Methanoculleus thermophilus* (*Methanomicrobiales*) [65]. It is uncertain whether this is the case also for *Ca*. Methanoregula pelomyxae, as we found two homologs of FdhA, but only one homolog of FdhB in the genome. Another possibility is the production of formate using the proton translocating formate hydrogenlyase complex. The genome contains a Hyf operon (METHP_1700-1705) composed of the hydrogenase and the membrane subunits of the formate hydrogenlyase complex. While we did not identify any FdhF in the genome that would interact with the formate hydrogenlyase complex, it could be that the second FdhA subunit might be involved in formate production using the formate hydrogenlyase complex.

The bacterial members of the consortium, *Ca*. Vesiculincola pelomyxae and *Ca*. Syntrophus pelomyxae, do not seem essential symbionts, as they can be removed by antibiotic treatment without affecting the viability of the host (Fig. S1). *Ca*. Vesiculincola pelomyxae represents an interesting case. Its metabolic capacity is very limited, nevertheless it retains critical pathways for biosynthesis of cell components and production of ATP via extended glycolysis. The major end products, acetate, CO_2_, and H_2_, are identical to those of the ameba (Fig. 4). At increasing H_2_ partial pressure, *Ca*. Vesiculincola pelomyxae would have to regenerate NAD via the Rnf complex, using the membrane potential generated at the expense of ATP. The bacterium also possesses an NTT, which might be involved in ATP/ADP translocation. ATP/ADP antiporters have been identified in various parasitic bacteria and eukaryotes [66–68]. Although it remains unclear how the nucleotides can pass through the host membrane, the expression values for the NTT in the transcriptomic analysis are higher than those of most enzymes involved in glycolysis (Dataset S3), suggesting that the amount of ATP obtained from the host may be substantial. In that case, the nature of the relationship between *Ca*. Vesiculincola pelomyxae and its host may be parasitic rather than commensal or mutualistic. This is also supported by observation that the bacterium lacks the capacity for the de novo biosynthesis of amino acids and nucleotides but possesses a large array of transporters that allow to take up amino acids and nucleosides from the host (Fig. 4). All these results taken together suggest that *Ca*. Vesiculincola pelomyxae is an intracellular parasite that depends on its host for survival. An interesting aspect of *Ca*. Vesiculincola pelomyxae is its capacity to produce bacteroicins. These small peptides, which have been shown to inhibit the growth of other closely related bacteria [69, 70], may serve to prevent colonization of the vacuole by other bacteria.

*Ca*. Syntrophus pelomyxae is a typical syntrophic bacterium with the capacity to ferment butyrate and isovalerate to acetate and H_2_. These pathways are thermodynamically unfavorable unless their end products are maintained at extremely low concentrations [49, 50]. Therefore, most bacteria that ferment fatty acids or other products of primary fermentations live in syntrophic associations with methanogens [71, 72], including representatives of *Syntrophobacterales* [73, 74]. Since butyrate and isovalerate are not predicted as products of the consortium, the most plausible source of these substrates is the external environment of the ameba, which should be rich in acetate, propionate, butyrate, isovalerate, and other products of anaerobic digestion [75, 76]. Potentially, isovalerate and butyrate may be produced also by the ameba during the digestion of proteins derived from phagocytized bacteria, as this would provide a source of amino acids. Although we did not directly observe digested bacteria in EM, it is very likely that *P. schiedti* feeds on bacteria, as phagocytic vesicles were observed in other strains of *P. schiedti* [2]. Accordingly, we identified two lysozyme genes encoded in the genome. Another source of energy for *Ca*. Syntrophus pelomyxae could be succinate that is produced by the MRO of *P. schiedti*. However, we think that the bacterium uses succinate for the biosynthesis of amino acids rather than for ATP synthesis (Fig. 7). This notion is based on the absence of a typical Sdh complex in the genome of *Ca*. Syntrophus pelomyxae. We only found a SdhAB operon, but it does not seem to be expressed (Dataset 4). A similar operon in the genome of *Syntrophobacter fumarioxidans* is not expressed during the growth on propionate in syntrophy with methanogens, while the canonical SdhABCD operons were highly expressed [71], suggesting a different, undocumented role for SdhAB.

The consortia of *P. schiedti* and *P. palustris* are quite similar with respect to the presence of a methanogen and a syntrophic deltaproteobacterium [19] (Figs. S5 and S6), and both of them are localized in the cytosol. Although we consider *Ca*. Syntrophus pelomyxae a commensal that benefits from the low hydrogen partial pressure provided by the methanogen [21, 48], the presence of similar symbionts in another *Pelomyxa* species suggests that it provides metabolic features that (although not essential) may increase the long-term fitness of its host.

The third partner in the consortia of the respective ameba, however, is unrelated and localized in different compartments. *Ca*. Vesiculincola pelomyxae (*Clostridia*) forms cell aggregates that are localized in membrane vesicles (Fig. 1C, D), whereas the *Rhodococcus* sp. (*Actinobacteria*) identified as the third symbiont of *P. palustris* is reportedly localized in the cytosol [19]. Since there are also reports of rod-shaped bacteria in *P. palustris* that are surrounded by a membrane of host origin [18], it remains possible that *P*. palustris harbors a symbiont that is located in a vesicle, but not aggregated. To better understand the role of the symbionts in the respective consortia, it will be necessary to investigate the diversity of the symbionts in additional *Pelomyxa* species and the consistency of the composition of the consortia in different strains of the same host species.

The study presented here elucidates the interactions within a complex quadripartite endosymbiotic community of a free-living anaerobic ameba. We show that *Ca*. Vesiculincola pelomyxae is most likely a parasite that benefits from the metabolic products of the host, while the *Ca*. Syntrophus pelomyxae oxidizes products of anaerobic digestion, most likely from the environment, in syntrophic association with the hydrogenotrophic methanogen *Ca*. Methanoregula pelomyxae. The latter is a mutualist that scavenges H_2_, acetate, and formate produced by the other members of the community and is essential for enabling the fermentative metabolism of both Ca. *Syntrophus polymyxae* and the ameba. Inhibition of methanogenesis results in the collapse of the consortium and the death of its members. In this respect the consortium resembles the syntrophic bacterial communities found in anaerobic digesters [77], and this has been suggested to be the case also in *P. palustris* [19]. The auxotrophies for amino acids and cofactors observed among the members of the consortium indicates sharing of nutrients and other potential interaction that are thought to contribute to the stability of other syntrophic communities under methanogenic conditions [78].

### Protologues

#### Candidatus Vesiculincola gen. nov

Ve.si.cul.in’co.la. L. fem. n. *vesicula*, a small bladder; L. masc. n. *incola*, inhabitant; N.L. masc. n. *Vesiculincola*, an inhabitant of vesicles.

The description is the same as for the type species, *Ca*. Vesiculincola pelomyxae.

#### Candidatus Vesiculincola pelomyxae sp. nov

pe.lo.my’xae. L. fem. dim. n. *vesicula*, a small bladder, vesicle; L. fem. n. *incola*, resident; N.L. fem. n. *vesiculincola*, resident of a vesicle. N.L. gen. sg. fem. n. *pelomyxae*, of *Pelomyxa*, a genus of amoebae, referring to the host.

Coccoid endospore-forming bacteria that occur endosymbiotically in vesicles of the genus *Pelomyxa* (Archamoebae). Hybridize with the specific oligonucleotide probe Rum-P-276 (Table S1). The genome accession number of the type strain is JARLUB000000000. Assigned to the family “Acutalibacteraceae” (*Clostridiales*) based on phylogenomic analysis (Fig. S4).

#### Candidatus Syntrophus pelomyxae sp. nov

pe.lo.my’xae. N.L. gen. sg. fem. n. *pelomyxae*, of *Pelomyxa*, a genus of amoebae, referring to the host.

Rod-shaped bacteria that occur endosymbiotically in the cytoplasm of the genus *Pelomyxa* (Archamoebae). Hybridize with the specific oligonucleotide probe Syn-P-182 (Table S1). The genome accession number of the type strain is JARLUC000000000. Assigned to the genus *Syntrophus* (*Deltaproteobacteria*) based on 16 S rRNA gene phylogeny (Fig. S5).

#### Candidatus Methanoregula pelomyxae sp. nov

pe.lo.my’xae. N.L. gen. sg. fem. n. *pelomyxae*, of *Pelomyxa*, a genus of amoebae, referring to the host.

Slender, rod-shaped archaea that occur endosymbiotically in the cytoplasm of the genus *Pelomyxa* (Archamoebae). Hybridize with the specific oligonucleotide probe Meth-P-972 (Table S1). The genome accession number of the type strain is CP121470. Assigned to the genus *Methanoregula* (*Methanomicrobiales*) based on 16 S rRNA gene phylogeny (Fig. S6).

### Supplementary information

Supplemental material & methods, supplementary figures S1–S6 and supplementary table S1–S3

Dataset 1

Dataset 2

Dataset 3

Dataset 4

Dataset 5

Supplemental Video S1

## Data Availability

The sequence data have been deposited in GenBank, https://www.ncbi.nlm.nih.gov/genbank, databases under National Center for Biotechnology Information (NCBI) BioProject PRJNA672820. The raw reads of the transcriptome sequencing have been deposited in the Sequence Read Archive (SRA), https://www.ncbi.nlm.nih.gov/sra (accession nos. SRR23919866– SRR23919877). The scaffolds assigned to the draft genome of “*Ca*. Vesiculincola pelomyxae”, draft genome of “*Ca*. Syntrophus pelomyxae” and draft genome of “*Ca*. Methanoregula pelomyxae” have been deposited in the NCBI genome database, https://www.ncbi.nlm.nih.gov/genome (accession nos. JARLUB000000000, JARLUC000000000, and CP121470, respectively).
