# Supplementary material for: Hydrogenotrophic methanogenesis is the key process in the obligately syntrophic consortium of the anaerobic ameba Pelomyxa schiedti
Source: ISME J. 2023 Aug 26;17(11):1884–94. doi: 10.1038/s41396-023-01499-6 (PMC10579272; doi:10.1038/s41396-023-01499-6)
Supplement: Supplementary file 1 — Supplemental material & methods, supplementary figures S1–S6 and supplementary table S1–S3 [file 41396_2023_1499_MOESM1_ESM.pdf]

Supplemental information

Materials and methods

Cell culture and manipulation

Polyxenic cultures of Pelomyxa *schiedti* strain SKADARSKE were maintained in Sonneborn's *Paramecium* medium [1] and transferred every two weeks as described previously [2]. Treatment of the culture with vancomycin (Sigma-Aldrich, USA) was performed at a final concentration of 40 µg/ml. For treatment of the culture with 2-bromoethanesulfonate (BES) (Sigma-Aldrich, USA) a final concentration of 5mM was used. In case of the cultures treated with antibiotics, the cells were passaged every week.

Sample preparation for FISH

Several tubes of Pelomyxa *schiedti* strain SKADARSKE were merged and pelleted by centrifugation at 600 x g for 10 minutes. The pelleted cells were resuspended in Trager U media [3] and samples were fixed on ice for six hours by adding three volumes of ice cold 4% formaldehyde in Trager U media. After fixation, the cells were pelleted by centrifugation at 600 x g for 10 minutes and washed three times with Trager U media. After the final wash, the pellet was resuspended in 50% ethanol:Trager U media. The fixed cells were stored at -20°C.

Probe design for 16S rRNA FISH

We used ARB 6.0.4 [4] and the Silva REF NR 99 SSU database release 126 for probe design (https://www.arb-silva.de/download/arb-files/). From the list of putative probe candidates generated by ARB we excluded probes which are located in regions with poor probe accessibility based on the *Escherichia coli* 16S rRNA accessibility map [5]. The designed probes were synthesized at Biomers.net (biomers.net GmbH, Germany) and labelled with various ATTO dyes at their 5' end. The probes used in this study are listed in Table S1.

Fluorescence in situ hybridization

FISH preparations were performed according to the protocol described in [6]. For all our probes, 30% formamide was used as optimal concentration. Hybridization of the probes was performed at 46°C for six hours then the slides were washed once at 48°C for 15 minutes. The slides were mounted using Vectashield mounting medium with DAPI (H-1200, Vector Laboratories). Images were acquired using a Leica SP8 confocal microscope, with gating enabled. Deconvolution of the acquired images was performed using Huygens Professional 22.10 and further processing was performed using ImageJ 1.53n.

Electron microscopy

Fixation of the cells was performed using 2.5% glutaraldehyde in 0.1 M cacodylate buffer as described previously [7]. The fixed cells were dehydrated in an ethanol series, transferred to acetone and then embedded in EPON resin. Ultrathin sections were prepared on an ultramicrotome (Reichert-Jung Ultracut E) with a diamond knife. Sections were stained with uranyl acetate and lead citrate and examined using a JEOL 1011 transmission electron microscope.

Transcriptome amplification

Transcriptome sequencing was performed based on a modified SmartSeq2 protocol [8] designed to capture also the bacterial transcripts. The modifications include bacterial cell lysis either using rLysozyme or Mutanolysin, polyadenylation of the transcripts using PolyA polymerase and blocking of the adenylation of rRNA using the EMBR-seq strategy [9]. EMBR primers were designed based on the 16S, 23S, 5S, for bacteria and 18S, 28S and 5.8S for the eukaryote. 20 µM of EMBR primer pools were prepared by mixing equimolar concentrations of the ordered EMBR primers (Table S2).

Briefly, a single cell of *Pelomyxa schiedti* was isolated by micromanipulation, washed two times in the same buffer, before being placed in a clear 0.2 ml PCR tube containing 0.1 µl of Recombinant RNAse inhibitor (Takara Bio, USA) and 0.9 µl of 0.2% Triton X-100 (Sigma-Aldrich, USA). The bacteria were lysed either with rLysozyme Solution (Merck Millipore, USA) or Mutanolysin (Sigma-Aldrich, USA). When rLysosyme was used, the stock solution of rLysozyme was diluted 100x using nuclease-free water. From the diluted rLysozyme, 0.7 µl were added to the isolated cell together with 0.3 µl of PolyA Polymerase buffer (M0276S, New England Biolabs, UK). When the bacteria were lysed with Mutanolysin, 0.5 µl of nuclease-free water, 0.2 µl of 1000U/mL Mutanolysin, and 0.3 µl PolyA Polymerase buffer were added to the picked cells. In both situations, the mixture was incubated at 37°C for 15 minutes. Meanwhile the adenylation mixture was prepared by mixing 1.25 µl of 20 µM EMBR primer pool, 1 µl of 10 mM ATP (New England Biolabs, UK), 1 µl of PolyA polymerase (New England Biolabs, UK) and 1.75 µl of nuclease-free water. After incubation 0.5 µl of adenylation mix was added to the reaction and the tube was incubated for 10 minutes at 37°C. Following incubation, 1 µl of 10 mM dNTP (Thermo Fisher Scientific, USA), 0.25 µl of 20 µM oligo dT and 0.25 µl of EMBR primer pool was added to the reaction then the tube was incubated at 65°C for five minutes to inactivate the PolyA polymerase followed by 58°C for one minute and 72°C for three minutes then the tube was placed immediately on ice. The downstream protocol followed directly the SmartSeq2 protocol starting from the "reverse transcription" step [8]. When the EMBR-seq primers were omitted, the primers volume was simply replaced by nuclease-free water. A total of 12 libraries were prepared: five libraries using rLysozyme as bacterial lysis reagent (three of these without EMBR primers, two with EMBR primers) and seven libraries using Mutanolysin (two of these without EMBR primers, five of them with EMBR primers).

Library preparation and sequencing

Transcriptome libraries were prepared using Nextera XT DNA Library preparation Kit (Illumina, San Diego, CA). Five of the prepared single-cell transcriptome libraries were sequenced on Illumina NovaSeq (PE

2x150bp) at Macrogen, Inc., South Korea, and seven of them were sequenced on Illumina NextSeq (PE 2x50bp) at Genomics Core Facility, EMBL, Heidelberg, Germany.

Genome assembly and binning

The single-cell genome assemblies were carried out using the seven single-cell genome sequencing experiments *P. schiedti* deposited previously [7] in NCBI as part of the Bio Project PRJNA672820. For each single-cell metagenome the raw reads were adapter and quality trimmed using Trimommatic 0.36 [10] with a quality threshold of 15. Afterwards the reads were assembled individually for each single-cell using SPAdes 3.13.0 [11] in single-cell (--sc) mode and with the k-mers of 21, 33, 55, 77, 99, and 121. From the assemblies, the eukaryotic data was removed by blastn against the *P. schiedti* reference genome (NCBI accession GCA_020536535.1). All scaffolds that had a significant hit (>90% identity and >80% coverage) towards the reference genome were classified as eukaryotic and removed. The remaining data was binned using the tetraESOM [12] method. Afterwards, the bins from individual single-cell metagenomes that contained identical small subunit rRNA (SSU) sequences were merged together and clustered further using cd-hit-est [13] at an identity level of 99%. Only the bins that were present in all seven single-cell assemblies were analyzed further. Using this approach, we generated five bins: a *Ca.* Methanoregula pelomyxae. bin, a Victivales bin, a *Ca*. Vesiculincola pelomyxae, *Ca.* Syntrophus pelomyxae, and an *Acetomicrobium* sp. bin. Each of the five bins were reassembled by mapping back the raw reads using BBMap from the BBTools package and taking just the mapped Illumina reads. Reassembly was performed using SPAdes 3.13.0 using the same parameters as described above. The reassemblies were performed both individually for each single-cell genome, as well as merged, by combining the reads for one bin from all seven single-cells. During reassembly we also added the Nanopore reads (accession no. SRX9527949) to improve the overall contiguity of the genomes. From the resulting assemblies, sequences below 500 bp were discarded. The bins were also checked for any potential contamination using a combination of blastn and blastp as described previously [14]. Finally, for each selected bin, a manual improvement of the assemblies was performed by aligning all generated assemblies to each other using MAUVE [15] and selecting the most contiguous scaffolds from each bin until all the unique sequences are included in the final assembly. In the case of *Ca*. Vesiculincola pelomyxae**,** the resulting assemblies were extremely fragmented due to extremely high genome coverage (>5000x). To improve the assemblies, the mapped reads were further normalized using BBNorm from the BBTools package, at an average coverage of 60x. The normalized reads were used to reassemble the genome and a manual improvement step was performed using MAUVE as described above.

Gap closure of *Ca.* Methanoregula pelomyxae chromosome using PCR

To circularize the assembly of *Ca.* Methanoregula pelomyxae, specific primers were designed (Table S3) based on the MAUVE alignment to amplify a missing portion of the genome for circularization. The amplification was carried out using Q5 DNA polymerase according to the manufacturer's protocol with an annealing temperature of 55°C for 30 seconds and extension at 72°C for 7 minutes. This resulted in a PCR product of ~8kbp that was cloned into pJET1.2 vector using CloneJET PCR Cloning Kit (Thermo Fisher

Scientific, USA) and sequenced in both directions using the primer walking strategy. The resulting DNA sequence was mapped back to the contig ends and used to circularize the archaeal chromosome.

Genome annotation

The annotation of the symbiotic bacterial genome sequences was performed using Prokka 1.14.6 automatic annotation pipeline [16]. This annotation was further completed using EggNOG-mapper [17, 18]. All the annotation sources were merged in a single GenBank file using emapper2gbk [19] and imported into Pathway Tools v 23.0 [20], for further curation and analysis. For all pathways of interest, the annotations were manually curated.

Phylogenetic analysis

For SSU rRNA gene phylogenies several datasets were created by downloading closely related sequences from NCBI. Where it was the case, the 16S rRNA sequences of the bacterial symbionts of *Pelomyxa palustris* [21] were also included in the datasets. The sequences were aligned using MAFFT 7 [22] with the G-INS-i algorithm followed by manual inspection. Trimming of the alignment was done using BMGE [23] using default parameters. Maximum Likelihood (ML) phylogenetic trees were constructed using IQ-TREE 1.6.12 [24] with the Model Finder Plus setting and with 1000 non-parametric bootstrap replicates.

For phylogenomic analysis of *Ca*. Vesiculincola pelomyxae, we used GTDB-tk [25] to generate a phylogenomic dataset from the 120 conserved bacterial genes. We used GTDB-tk identify to get the single copy genes from the genome of *Ca*. Vesiculincola pelomyxae and GTDB-tk align to align these genes to representatives already present in the GTDB database [26]. We limited the database to the families Acutalibacteraceae, Ruminococcaceae and Ethanoligenenaceae from the order Oscilospiralles. The aligned and untrimmed dataset generated by GTDB-tk align, was further refined to keep at least one representative for each genus from the dataset. The alignment was trimmed using BMGE, with default parameters. The final alignment contained 230 taxa and 27340 amino acid positions. A ML phylogenetic tree was inferred by IQ-TREE 1.6.12 using the Posterior Mean Site Frequency (PMSF) empirical model with a LG+F+G guide tree. The branch supports were estimated using the ultrafast bootstrapping strategy with 10000 bootstrap replicates.

Expression analysis

Raw reads generated by transcriptome sequencing were adapter and quality trimmed using fastp [27]. The trimmed reads were mapped and separated by BBSplit from the BBTools package, using the four genomes (*Pelomyxa schiedti*, *Ca.* Vesiculincola pelomyxae, *Ca.* Methanoregula pelomyxae, and *Ca.* Syntrophus pelomyxae) as references. The mapped reads for each genome were merged together from all seven transcriptomes and mapped back to their respective genomes using Bowtie 2 [28]. FeatureCounts [29] was used to assign each read to gene features. The read count was used to calculate Transcripts Per Million (TPM) for each gene using a python script. TPM was used to evaluate expression levels of the genes for each organism.

References

1. Sonneborn TM. Methods in the general biology and genetics of *Paramecium aurelia*. *J Exp Zool* 1950; **113**:87–147.

2. Zadrobílková E, Walker G, Čepička I. Morphological and molecular evidence support a close relationship between the free-living Archamoebae *Mastigella* and *Pelomyxa*. *Protist* 2015; **166**:14–41.

3. Trager W. The cultivation of a cellulose-digesting flagellate, *Trichomonas termopsidis*, and of certain other termite protozoa. *Biol Bull* 1934; **66**:182–190.

4. Ludwig W, Strunk O, Westram R, Richter L, Meier H, Yadhukumar, et al. ARB: a software environment for sequence data. *Nucleic Acids Res* 2004; **32**:1363–1371.

5. Behrens S, Fuchs BM, Mueller F, Amann R. Is the in situ accessibility of the 16S rRNA of *Escherichia coli* for Cy3-labeled oligonucleotide probes predicted by a three-dimensional structure model of the 30S ribosomal subunit? *Appl Environ Microbiol* 2003; **69**:4935–41.

6. Manz W, Amann R, Ludwig W, Wagner M, Schleifer K-H. Phylogenetic oligodeoxynucleotide probes for the major subclasses of Proteobacteria: problems and solutions. *Syst Appl Microbiol* 1992; **15**:593–600.

7. Záhonová K, Treitli SC, Le T, Škodová-Sveráková I, Hanousková P, Čepička I, et al. Anaerobic derivates of mitochondria and peroxisomes in the free-living amoeba *Pelomyxa schiedti* revealed by single-cell genomics. *BMC Biol* 2022; **20**:56.

8. Picelli S, Faridani OR, Björklund AK, Winberg G, Sagasser S, Sandberg R. Full-length RNA-seq from single cells using Smart-seq2. *Nat Protoc* 2014; **9**:171–181.

9. Wangsanuwat C, Heom KA, Liu E, O'Malley MA, Dey SS. Efficient and cost-effective bacterial mRNA sequencing from low input samples through ribosomal RNA depletion. *BMC Genomics* 2020; **21**:717.

10. Bolger AM, Lohse M, Usadel B. Trimmomatic: a flexible trimmer for Illumina sequence data. *Bioinformatics* 2014; **30**:2114–20.

11. Bankevich A, Nurk S, Antipov D, Gurevich AA, Dvorkin M, Kulikov AS, et al. SPAdes: a new genome assembly algorithm and its applications to single-cell sequencing. *J Comput Biol* 2012; **19**:455–477.

12. Haddad I, Hiller K, Frimmersdorf E, Benkert B, Schomburg D, Jahn D. An emergent self-organizing map based analysis pipeline for comparative metabolome studies. *In Silico Biol* 2009; **9**:163–178.

13. Li W, Godzik A. Cd-hit: a fast program for clustering and comparing large sets of protein or nucleotide sequences. *Bioinformatics* 2006; **22**:1658–9.

14. Treitli SC, Kolisko M, Husník F, Keeling PJ, Hampl V. Revealing the metabolic capacity of *Streblomastix strix* and its bacterial symbionts using single-cell metagenomics. *Proc Natl Acad Sci U S A* 2019; **116**:19675–19684.

15. Darling AE, Mau B, Perna NT. progressiveMauve: Multiple genome alignment with gene gain, loss and rearrangement. *PLoS One* 2010; **5**:e11147.

16. Seemann T. Prokka: rapid prokaryotic genome annotation. *Bioinformatics* 2014; **30**:2068–2069.

17. Cantalapiedra CP, Herṇandez-Plaza A, Letunic I, Bork P, Huerta-Cepas J. eggNOG-mapper v2: Functional annotation, orthology assignments, and domain prediction at the metagenomic scale. *Mol Biol Evol* 2021; **38**:5825–5829.

18. Huerta-Cepas J, Szklarczyk D, Heller D, Hernández-Plaza A, Forslund SK, Cook H, et al. eggNOG 5.0: a hierarchical, functionally and phylogenetically annotated orthology resource based on 5090 organisms and 2502 viruses. *Nucleic Acids Res* 2019; **47**:D309–D314.

19. Belcour A, Frioux C, Aite M, Bretaudeau A, Hildebrand F, Siegel A. Metage2metabo, microbiota-scale metabolic complementarity for the identi1cation of key species. *Elife* 2020; **9**:e61968.

20. Karp PD, Midford PE, Billington R, Kothari A, Krummenacker M, Latendresse M, et al. Pathway Tools version 23.0 update: software for pathway/genome informatics and systems biology. *Brief Bioinform* 2021; **22**:109–126.

21. Gutiérrez G, Chistyakova L V., Villalobo E, Kostygov AY, Frolov AO. Identification of *Pelomyxa palustris* endosymbionts. *Protist* 2017; **168**:408–424.

22. Katoh K, Standley DM. MAFFT multiple sequence alignment software version 7: improvements in performance and usability. *Mol Biol Evol* 2013; **30**:772–780.

23. Criscuolo A, Gribaldo S. BMGE (Block Mapping and Gathering with Entropy): a new software for selection of phylogenetic informative regions from multiple sequence alignments. *BMC Evol Biol* 2010; **10**:210.

24. Nguyen L-T, Schmidt HA, von Haeseler A, Minh BQ. IQ-TREE: a fast and effective stochastic algorithm for estimating maximum-likelihood phylogenies. *Mol Biol Evol* 2015; **32**:268–274.

25. Chaumeil PA, Mussig AJ, Hugenholtz P, Parks DH. GTDB-Tk: a toolkit to classify genomes with the Genome Taxonomy Database. *Bioinformatics* 2020; **36**:1925–1927.

26. Parks DH, Chuvochina M, Chaumeil PA, Rinke C, Mussig AJ, Hugenholtz P. A complete domain-to-species taxonomy for Bacteria and Archaea. *Nat Biotechnol 2020 389* 2020; **38**:1079–1086.

27. Chen S, Zhou Y, Chen Y, Gu J. fastp: an ultra-fast all-in-one FASTQ preprocessor. *Bioinformatics* 2018; **34**:i884–i890.

28. Langmead B, Salzberg SL. Fast gapped-read alignment with Bowtie 2. *Nat Methods* 2012; **9**:357–359.

29. Liao Y, Smyth GK, Shi W. featureCounts: an efficient general purpose program for assigning sequence reads to genomic features. *Bioinformatics* 2014; **30**:923–930.

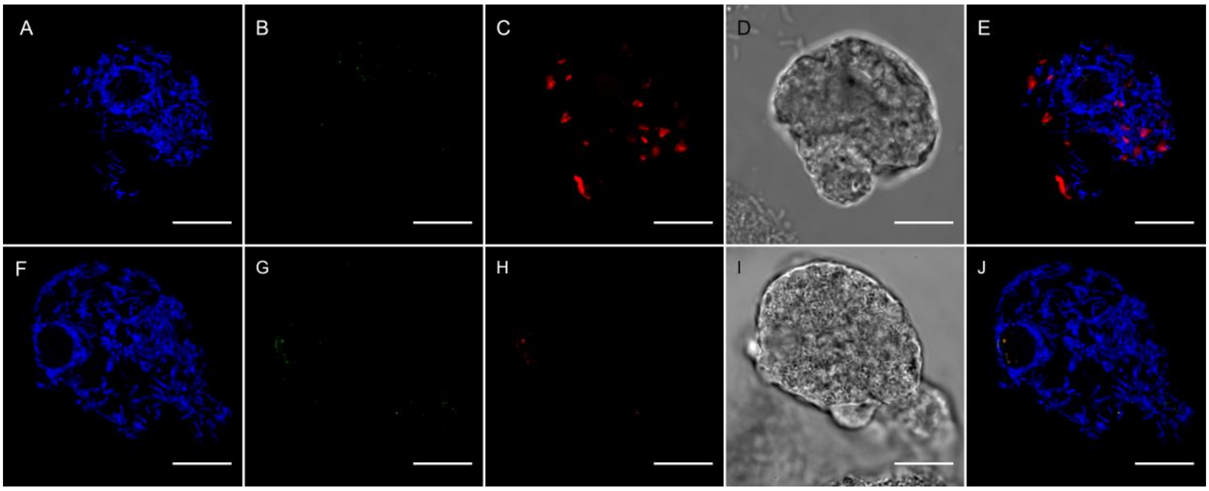

Figure S1. FISH identification of the bacterial symbionts present in *Pelomyxa schiedti* six weeks (A-E) and four months (F-J) of vancomycin treatment: **A**, **F** – Localisation of *Ca.* Methanoregula pelomyxae using the Meth-P-972 probe; **B**, **G** – Localisation of *Ca.* Vesiculincola pelomyxae using Rum-P-276 probe; **C**, **H** – Localisation of *Ca.* Syntrophus pelomyxae using the SYNP-P-182 probe; **D, I** – DIC view of the cell; **F** - Merged picture of **A**, **B** and **C**; **J** -Merged picture of **F**, **G** and **H**; All scale bars represent 10 µm.

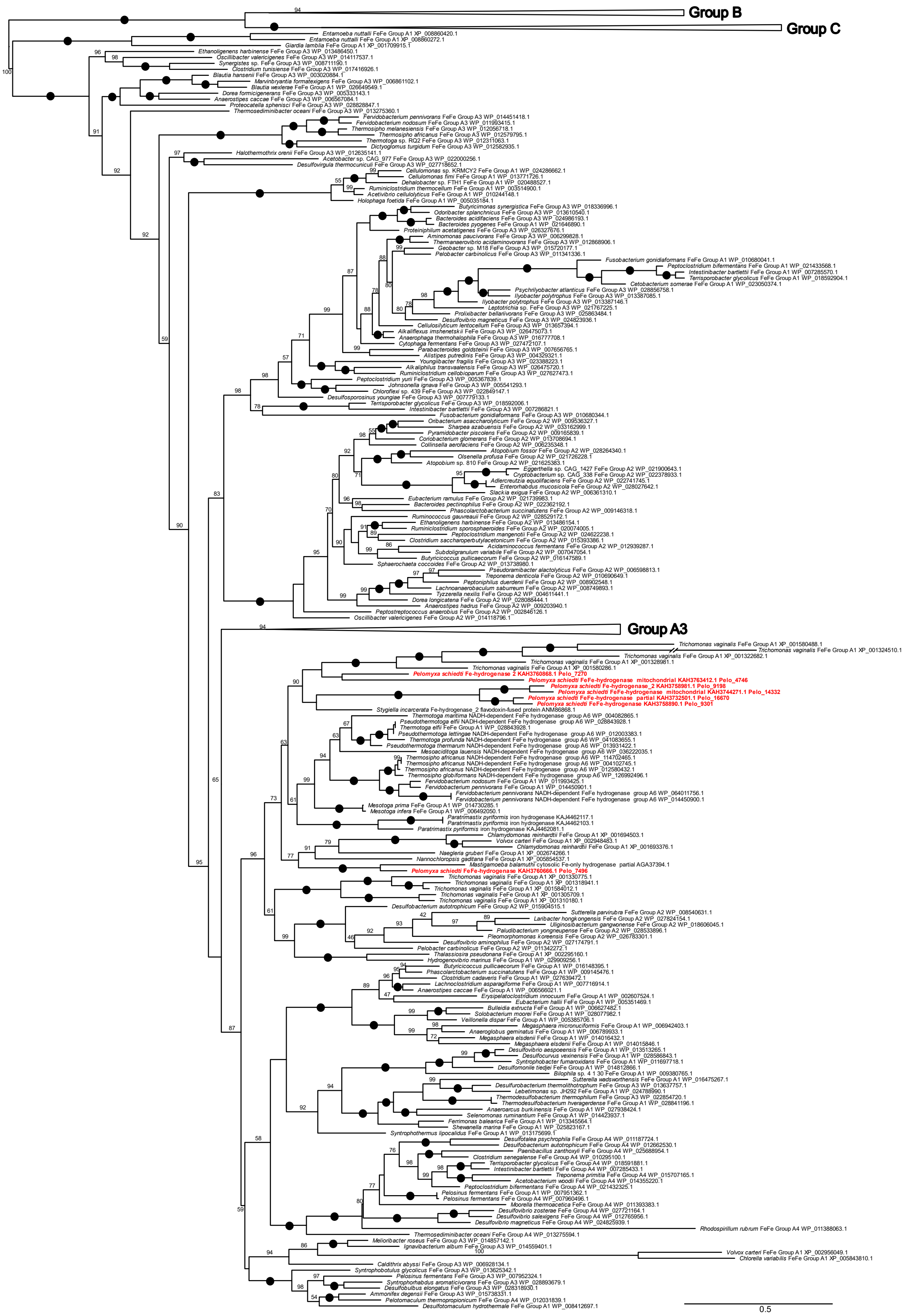

Figure S2. Maximum likelihood (ML) tree showing the relationship of *Pelomyxa schiedti* hydrogenases. The tree was constructed using IQ-tree based on 188 aligned sites. Values at the nodes represent ML bootstrap support values from 10000 ultrafast bootstrap replicates. Dots represent full support. Values below 50 are not shown. Scale bar corresponds to 0.5 expected substitutions per site.

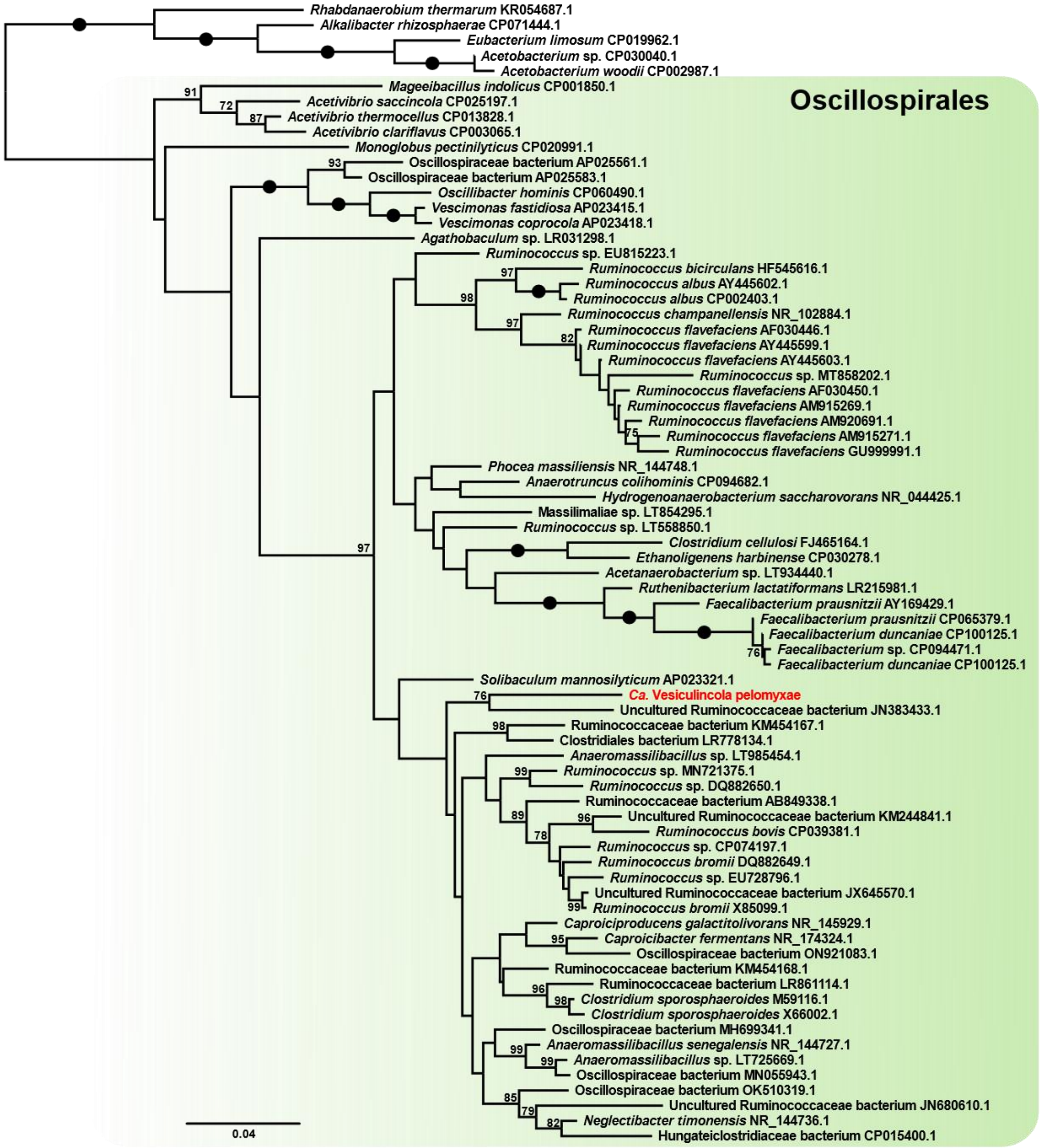

Figure S3. Maximum likelihood (ML) tree showing the relationship of *Ca.* Vesiculincola pelomyxae within Oscilospiralles. The tree was constructed using IQ-tree based on 1348 aligned sites. Values at the nodes represent ML bootstrap support values from 1000 non-parametric bootstrap replicates. Dots represent full support. Values below 70 are not shown. The tree was rooted with representatives of Eubacteriales. Scale bar corresponds to 0.04 expected substitutions per site.

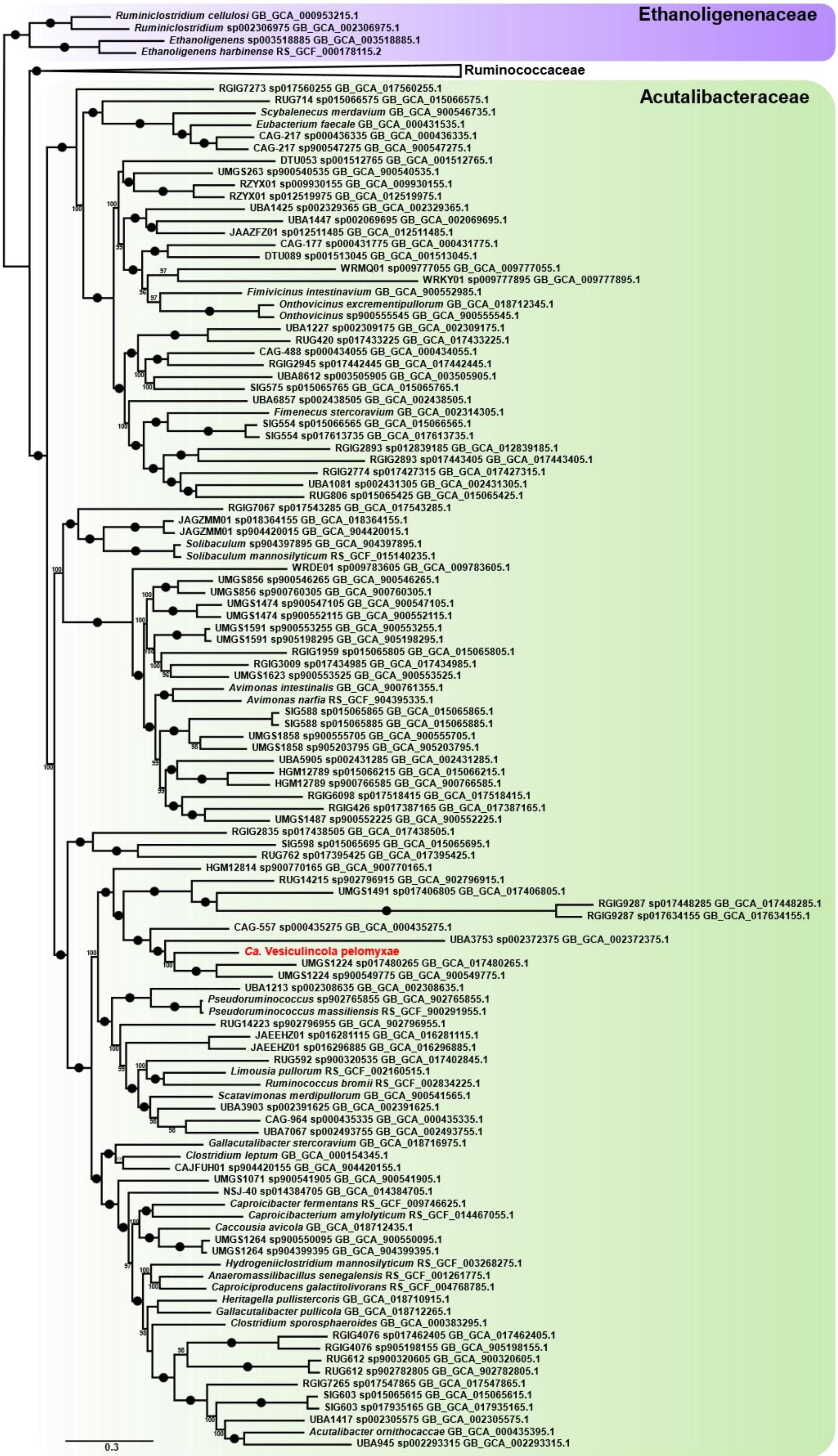

Figure S4. Maximum likelihood (ML) tree showing the position of *Ca.* Vesiculincola pelomyxae in the order Oscilospiralles. The tree was constructed using IQ-tree based on 27340 aligned sites. Values at the nodes represent ML bootstrap support values from 10000 ultrafast bootstrap replicates. Dots represent full support. Values below 95 are not shown. The tree was rooted with representatives of Ethanoligenenaceae. Scale bar corresponds to 0.3 expected substitutions per site.

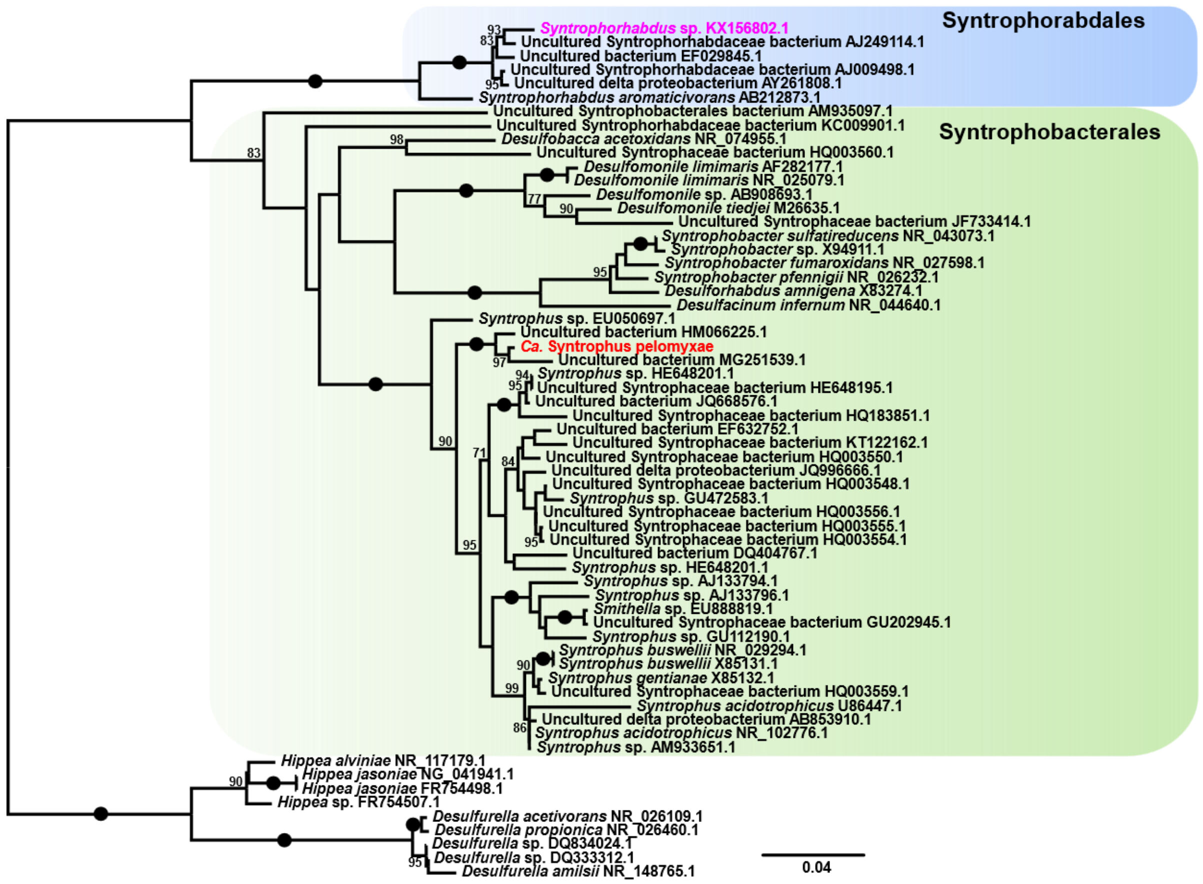

Figure S5. Maximum likelihood (ML) tree showing the relationship of *Ca.* Syntrophus pelomyxae within Syntrophobacterales. The tree was constructed using IQ-tree based on 938 aligned sites. Values at the nodes represent ML bootstrap support values from 1000 non-parametric bootstrap replicates. Dots represent full support. Values below 70 are not shown. The tree was rooted with representatives of Desulfurellales. The identified endosymbiont of *P. palustris* is highlighted in purple. Scale bar corresponds to 0.04 expected substitutions per site.

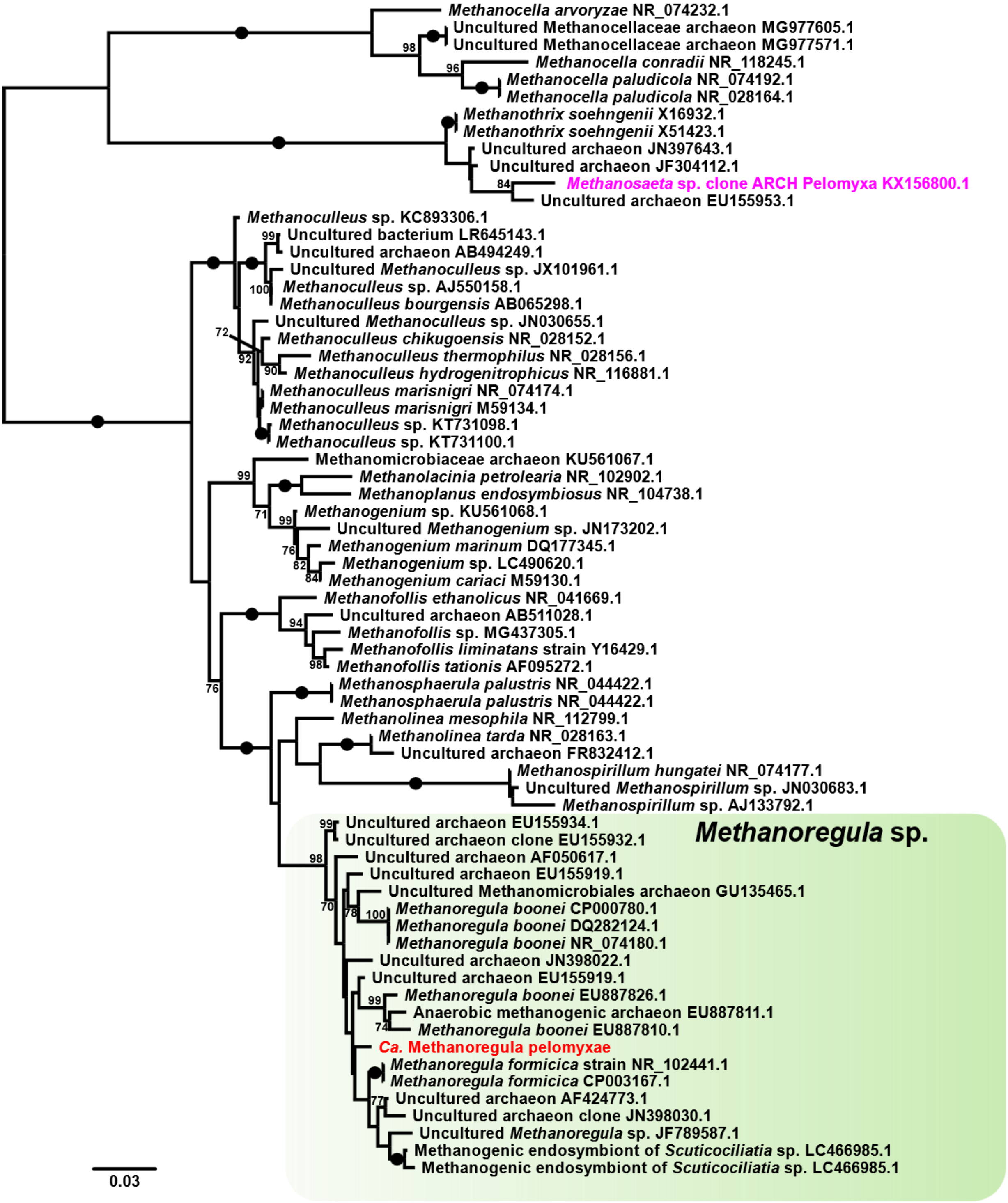

Figure S6. Maximum likelihood (ML) tree showing the relationship of *Ca.* Methanoregula pelomyxae within Methanomicrobiales. The tree was constructed using IQ-tree based on 1360 aligned sites. Values at the nodes represent ML bootstrap support values from 1000 non-parametric bootstrap replicates. Dots represent full support. Values below 70 are not shown. The tree was rooted with representatives of *Methanocellales*. The identified endosymbiont of *P. palustris* is highlighted in purple. Scale bar corresponds to 0.03 expected substitutions per site.

Table S1. List of the probes and their sequences used for identification of symbiotic bacteria.

Probe Name	Probe sequence (5'-3')	Optimal formamide concentration	Targeted organism
Rum-P-276	ACCCGGCTACCGATCGTAGC	30%	*Ca*. Ruminococcus pelomyxae
Syn-P-182	CAGCCGAGGCCATGCAGCGT	30%	*Ca*. Syntrophus pelomyxae
Meth-P-972	CTAACTGGTGAGTTTTCCGG	30%	*Ca*. Methanoregula pelomyxae
Vic-P-175	CGTTCAGGAGCCACATTCGG	20%	*Victivales sp.*
Ace-P-85	CGCCACTTTCTAAAGGTGCA	30%	*Acetomicrobium*

Table S2: List of EMBR primers designed to block rRNA adenylation.

Name	Primer sequence (5’-3’)
METH_5S	AGCAGCTTGGTATAGTTCCCGGGGAC
METH_16S	AGGAGGTGATCCAGCCGCAGATTCCCC
METH_23S	GGACGTTAGTACTTCCGGACTGAAC
PELO_5S	CACAGCGCTCCCAGGTAATCCCTTAC
PELO_18S	TGATCCTTCCGCAGGTTCACCTACGG
Pelo_28S_1	GCATCTCTCGGCCGGGCCTGCCTTGG
Pelo_28S_2	CAGGTGTCCTAAGAGTGGCTCATAGAGAAC
PELO_58S	GGAGCGCAGATTGCATTCAAAATTTC
RUM_5S	TGGCATCTTCCTATTTTCCCAGGCCG
RUM_16S	AAAGGAGGTGATCCAGCCGCACCTTCCG
RUM_23S	GGTCAAGCCCTCGACCTATTAGTACTGCC
SYNP_5S	GGCGGCGACCTACTCTCCCACACAGTC
SYNP_16S	AAAGGAGGTGATCCAGCCACAGGTTCCC
SYNP_23S	TGGTCAAGCCTCACGGCCGATTAGTATCAG

Table S3: List of primers used in this study.

Name	Primer sequence (5’-3’)
Meth_GP_F	GCTCTCTCATCAATCATTCGGAACG
Meth_GP_R	GCCGGGATATATCTTTTGAGGAACGC
